# Multifocal subdural hematomas as the presenting sign of acquired hemophilia A: a case report

**DOI:** 10.1186/1756-0500-7-134

**Published:** 2014-03-08

**Authors:** Mark J Burish, Aimee Aysenne, Vineeta Singh

**Affiliations:** 1 Department of Neurology, University of California, San Francisco, USA; 2 505 Parnassus Ave, Room M-798, Box 0114, San Francisco, CA 94143-0114, USA

**Keywords:** Acquired hemophilia A, Nontraumatic subdural hematoma, Intracranial hemorrhage, Secondary headache syndrome, Lupus, Spinal cord, Critical care

## Abstract

**Background:**

Acquired hemophilia A (AHA) is a rare coagulopathy linked to a variety of etiologies including autoimmune diseases, neoplasms, diabetes, respiratory diseases, and the post-partum state. While bleeding in AHA is often seen in mucocutaneous or intramuscular locations, intracranial and intraspinal bleeds are exceedingly rare.

**Case presentation:**

We report an unusual case of spontaneous multifocal subdural hematomas in a 25 year old Asian woman with lupus who presented with headache and backache, and was found to have an elevated partial thromboplastin time (PTT) level and new diagnosis of AHA.

**Conclusions:**

Subdural hematomas as the initial sign of AHA are all but unknown in the medical literature. We bring this entity to the attention of the neurology community because lumbar puncture and/or conventional angiogram are often indicated in the work-up of idiopathic multifocal subdural hematomas, but may be dangerous in patients with AHA.

## Background

Acquired hemophilia A (AHA) is a rare disorder (approximately 1 in 1 million persons per year) but carries a significant mortality of 8–22% [1]. While bleeding in AHA most commonly involves mucocutaneous or intramuscular locations, it can also involve the gastrointestinal or genitourinary tracts [2]. Hemarthrosis, which is considered a hallmark of congenital Hemophilia, is rarely encountered in AHA. While intracranial hemorrhage has been well documented in congenital hemophilia A [3-6], there are only a limited number of publications on intracranial bleeds in acquired hemophilia A [7-10], suggesting that it is a very rare phenomenon. Diagnosis is made based on the finding of 1) an elevated partial thromboplastin time (PTT) which does not correct after 2 hours of mixing with normal plasma (mixing study), 2) no administration of heparin, which can be determined by a prolonged thrombin time but normal reptilase time, 3) negative testing for lupus anticoagulants, and 4) the presence of an inhibitor as shown on the Bethesda assay, which mixes serial dilutions of the patient’s blood with normal plasma [11,12]. The etiology is idiopathic in 50% and post-partum in 10%, with the remaining 40% covering a wide spectrum of autoimmune, allergic, neoplastic, diabetic, and respiratory diseases [11,13]. Treatment in part depends on the underlying etiology of the AHA, with pregnancy- or allergy-related AHA often responding to steroids or cyclophosphamide and resolving spontaneously, tumor-related AHA often depending on treatment of the malignancy, and autoimmune-related AHA often requiring stronger immunosuppression and rarely resolving spontaneously [13].

## Case presentation

A 25 year old Asian woman with a history of lupus presented to the emergency room with headaches not responding to over the counter analgesics. The headaches started 6 days prior, with no reported history of trauma. She described the headaches as a gradual onset of intermittent 10/10 sharp right-sided head pain which lasted for several seconds only, and occurred multiple times per day. The pain occasionally spread to the right neck with a pulsating quality but without tinnitus. There was no postural component, no associated migrainous features (including nausea, vomiting, photophobia, phonophobia, osmophobia, or vision changes), and no trigeminal autonomic symptoms (including facial pallor, facial flushing, lacrimation, or rhinorrhea). In addition, she occasionally had brief lower back spasmic-type pain with no radiation. She did not have any symptoms of coagulopathy including easy bleeding, GI bleeds, epistaxis, or hemoptysis. She carried a diagnosis of lupus with discoid rash and arthralgia as predominant symptoms for which she was taking prednisone 15 mg daily and mycophenylate 500 mg BID (she had self-discontinued the mycophenylate 1 week prior to admission), and a family history significant for a sister who died of anemia of an unknown etiology at the age of 24. Our patient’s neurologic examination was unremarkable.

**Figure 1:**
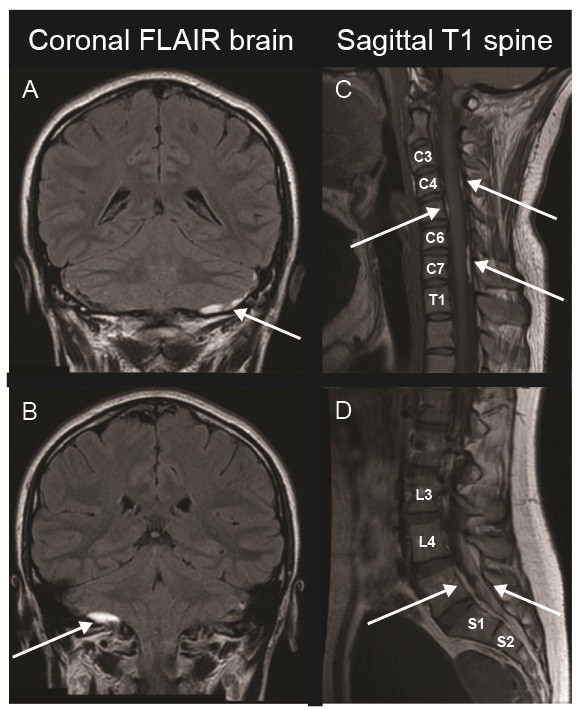
**Diffuse spontaneous subdural hematoma associated with acquired hemophilia A.** MRI showing coronal brain FLAIR sequence **(A and B)**, sagittal T1 sequence of the cervical spine **(C)** and sagittal T1 sequence of the lumbar spine **(D)** demonstrating multiple subdural hematomas indicated by arrows. The patient had subdural hematomas located in the bilateral infracerebellar, cervical (C1-C7, maximal at C6-7), and lumbar (L4-S1) regions.

Given the new onset of severe unilateral headaches without clear symptoms of a primary headache syndrome, head imaging was performed. Brain MRI revealed bilateral infracerebellar and right pre-pontine extra-axial collections most consistent with hematomas (Figure 1A and B), and basic laboratory studies were significant for an isolated elevation of PTT at 74 with a normal CBC, chemistry panel, liver panel, INR, and urine toxicology screen. Additional imaging included a brain MR venogram with no venous thrombosis, a CT angiogram with no vasculitis or aneurysms, and a spinal MRI with additional subdural hematomas in her cervical and lumbar regions (Figure 1C and D). Work-up of her elevated PTT revealed a clotting mixing study that failed to correct with a 1:1 ratio to plasma (suggestive of the presence of an inhibitor) and an undetectable factor 8 level, consistent with AHA (one month after treatment the factor 8 activity level was 36% with a normal range of 56–191%). Lupus anticoagulant testing had previously been performed twice over 6 months, with an initial indeterminate anti-cardiolipin IgG of 17 that was normal at 10 on rechecking, and normal levels of anti-cardiolipin IgM, anti-beta-2-glycoprotein IgG, anti-beta-2-glycoprotein IgM, Russell Viper Venom Test, and Russell Viper Venom confirmation testing. Urine pregnancy testing was negative and she was not post-partum. Infectious labs revealed a positive beta-D-glucan and negative galactomannan, but a brochoalveolar lavage that was positive only for candida and rare gram-positive cocci and was negative for acid fast bacilli, other fungi, and viral cultures. Other infectious testing was negative including a nasopharyngeal swab for influenza A and B, urine cultures, blood cultures, histoplasma antigen, and a hepatitis panel. She was started on recombinant factor 7a and rituximab, with repeat imaging showing resolving hematomas.

## Conclusions

Of the multiple etiologies of AHA [11,13], our patient’s disease was most likely related to her systemic lupus erythematosus. Our patient’s headache and backache description, specifically the very brief and intermittent nature of her symptoms, is unusual for pain related to mass lesions. One previous case report of subdural hematomas and AHA described more classic symptoms of headache and drowsiness [7], but given the paucity of cases in the literature, it is difficult to say if there is any characteristic headache associated with AHA.

On initial work-up of our patient, a variety of diagnostic tests were discussed. The differential diagnosis for non-traumatic intracranial and intraspinal subdural hematomas is broad, and etiologies include neoplasms such as myelodysplastic disorders [14] or meningiomas [15], infections such as mycotic aneurysms [16], vascular diseases such as arteriovenous malformations [17], aneurysms [18], or primary CNS vasculitis [19], and coagulopathies such as idiopathic thrombocytopenic purpura [20]. Thus a thorough investigation for non-traumatic subdural hematoma may lead to a lumbar puncture or a conventional angiogram. The risk of bleeding after lumbar puncture is not known in patients with AHA, or even for similar procedures such as epidural injections in more common coagulopathies [21]. However the risk of bleeding after lumbar puncture is presumably higher for patients with untreated AHA than for the general population. Before performing a lumbar puncture on patients who are otherwise stable, providers might consider initiating treatment or awaiting results of the AHA testing. Similarly conventional angiogram may pose unnecessary risks in patients with AHA, and deferral or avoidance of angiogram should also be considered, especially if other vessel imaging such as MRA or CTA is not suggestive of vascular malformations.

**Figure 2:**
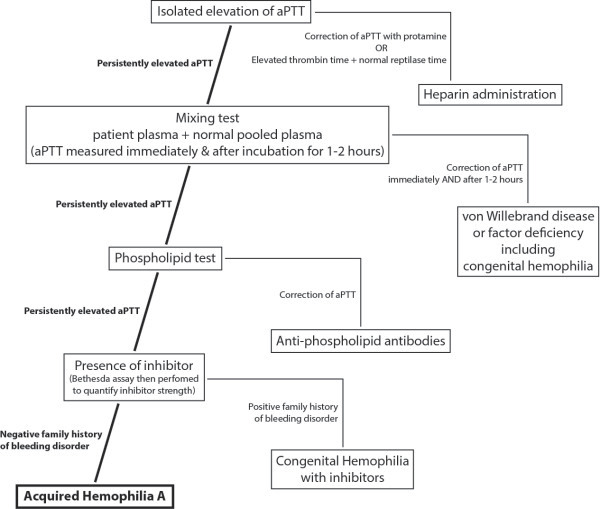
**Algorithm for the diagnosis of acquired hemophilia A.** Based on the response to mixing tests and phospholipid tests, acquired hemophilia A can be distinguished from other disorders causing an isolated elevation of aPTT.

The findings of subdural hematoma with an unexplained elevated PTT should prompt further work-up for AHA with mixing studies at 0.1 and 2 hours, phospholipid studies for lupus anticoagulant, and the Bethesda assay for the quantification of inhibitors (Figure 2) [11,12,22]. In patients with suspected AHA with any pain complaint, such as lower back pain in our case, imaging should be considered to look for additional bleeds and to lend stronger support for starting immediate factor replacement therapy. In the acute setting, if emergent neurosurgical evacuation of these patients is required, factor 7a should be considered for uncontrolled intra-operative bleeding.

## Consent

Written informed consent was obtained from the patient for publication of this Case report and any accompanying images. A copy of the written consent is available for review by the Editor of this journal.

## Competing interests

The authors (M.J. Burish, A. Aysenne, and V. Singh) declare that they have no competing interests.

## Authors’ contributions

MJB contributed by drafting and revising the manuscript for content, and analysis/interpretation of data. He also assisted in acquisition of data. AA contributed by revising the manuscript for content, and analysis/interpretation of data. She also assisted in acquisition of data. VS contributed by revising the manuscript for content, and analysis/interpretation of data. She also assisted in acquisition of data. All authors read and approved the final manuscript.
